# Evaluation of Clinical Practice Guidelines for Rare Diseases in Japan

**DOI:** 10.31662/jmaj.2022-0094

**Published:** 2022-09-30

**Authors:** Tomoe Uchida, Yoshimitsu Takahashi, Hiromitsu Yamashita, Yuriko Nakaoku, Tomoko Ohura, Takashi Okura, Yuko Masuzawa, Masayoshi Hosaka, Hiroshi Kobayashi, Tami Sengoku, Takeo Nakayama

**Affiliations:** 1 Department of Health Informatics, Kyoto University School of Public Health, Kyoto, Japan; 2 Department of Preventive Medicine and Epidemiology, National Cerebral and Cardiovascular Center, Suita, Japan; 3 Division of Occupational Therapy, Department of Rehabilitation, Faculty of Health Sciences, Naragakuen University, Nara, Japan; 4 Department of Health and Welfare, Graduate School of Health and Welfare Science, Okayama Prefectural University, Soja, Japan; 5 St. Luke’s International University, Tokyo, Japan

**Keywords:** Clinical practice guideline, rare diseases, evidence-based medicine, informed decision-making, non-medical resources, illnesses

## Abstract

**Introduction:**

The insufficient quantity and quality of clinical epidemiological evidence in the field of rare diseases have posed methodological challenges to develop clinical practice guidelines (CPGs). Guideline development groups struggle to provide patients and their families with beneficial guidance, such as that for medical care and in complex circumstances. Motivated by the challenges, we focused on information on resources for supporting the daily and social life to improve the CPGs for users. We aimed to assess the methodological quality of CPGs for rare diseases in Japan and to evaluate information on resources to support the daily and social life in the CPGs.

**Methods:**

We conducted a systematic search using PubMed, three electronic Japanese databases, and two hand-searched sources in Japan. The Appraisal of Guidelines for Research and Evaluation (AGREE) II instrument with six domains was used to assess the methodological quality of the CPGs. A content analysis of the CPG text was conducted using five keywords as information on non-medical resources, e.g., “Intractable Disease Consultation Support Center,” “Japan Intractable Disease Information Center,” and “Patient Association.”

**Results:**

A total of 55 CPGs met the inclusion criteria. Among four domains of AGREE II with low scores (Stakeholder Involvement, Rigor of Development, Applicability, and Editorial Independence), Rigor of Development had the lowest median score. As for information on non-medical resources, 41 CPGs included at least 1 of the 5 keywords, while 14 CPGs included none.

**Conclusions:**

At the Rigor of Development domain, methodological challenges may have resulted in an insufficient description of items regarding the translation evidence to recommendations. As the sufficiency of five keywords as information on non-medical resources could be improved, the information will be advocative as clues to provide pragmatic guidance, particularly for rare diseases with limited medical evidence.

## Introduction

The World Health Organization states that rare diseases are a global health issue. It is roughly estimated that 400 million people worldwide are affected by a rare disease ^(1)^. In recent decades, systematic global efforts for entire rare diseases have led to improvements in the duration of survival and the quality of life of patients living with rare diseases. Various challenges that need further support remain, including the implementation of guidelines for medical and psychosocial care for rare diseases ^(1)^. Orphanet is a globally recognized reference source for rare diseases and expertise to ensure equal access to knowledge for all stakeholders ^(2)^. It has been developed in Europe since 1997 and provides clinical practice guidelines (CPGs) ^(2)^. Although people living with rare diseases are psychologically, socially, economically, and culturally vulnerable, Orphanet mentions that these difficulties can be overcome with appropriate policies ^(2)^.

Insufficient quantity and quality of clinical evidence have posed methodological challenges to develop CPGs for rare diseases ^(3)^. The RARE-Bestpractices Working Group concluded that the GIN-McMaster Guideline Development Checklist and the GRADE methodology are both applicable in the field of rare diseases ^(4)^. In particular, according to this group, the “evidence to decision” table is suitable for determining the recommendations made for rare diseases ^(4), (5), (6)^. Although adopting these methodologies is the best course of action currently, low or very low quality of the evidence generally leads to weak recommendations ^(6)^. Moreover, the barriers to generating evidence, such as difficulties in conducting sufficiently large studies on rare diseases with minimal bias, have still limited certainty of evidence ^(3), (6)^.

European Reference Networks (ERNs) develop and implement CPGs with a rigorous and distinct methodology, including expert consensus for psychosocial support and social care considerations ^(7)^. ERNs deal with complex conditions or rare diseases that require highly specialized treatment and a concentration of knowledge and resources ^(8), (9)^. ERNs have mentioned that “CPGs serve as a great equalizer in the field of rare diseases: as a matter of fact, they can mean the difference between no care/substandard care and patients living longer, healthier lives with fewer complications ^(10)^.” Furthermore, they have emphasized developing care pathways to address the challenges faced by people with rare diseases, including long diagnostic odysseys, limited and unequal access to treatments, a heavy burden of complex care coordination, and difficulties in social life ^(11), (12), (13)^.

In Japan, the Act on Medical Care for Patients with Rare/Intractable Diseases (Act No. 50 of 2014, Rare/Intractable Diseases Act) ^(14)^ was enforced in January 2015. Its essential principles are research-based care to overcome rare and intractable diseases, financial support for receiving health and social care, and a society where people with rare and intractable diseases and their families can live with dignity in the community ^(15)^. The Act lays out the care pathways framework for people living with rare and intractable diseases, including infrastructure for access to diagnostics and resources, to provide a high level of health and social care ^(16)^. Since 2014, evidence-based development of CPGs for rare and intractable diseases is required in Japan ^(17), (18), (19)^. However, guideline development groups struggle to provide patients and their families with beneficial guidance, such as that for medical care and in complex circumstances, in evidence-limited situations ^(20)^. Rare diseases impact physically, emotionally, and socially on affected individuals as their condition progresses deepening their sense of loss in the context of their daily lives ^(21)^. Psychosocial care is required to support the daily and social lives of patients with rare diseases. To address these challenges, organizing care pathways and bridging the gap between health, social, and local support/services are essential ^(22), (23)^. Therefore, CPGs that provide information on the resources available to patients with rare diseases could help patients, caregivers, and practitioners more than those based only on clinical evidence.

This study aimed to assess the methodological quality of CPGs for rare diseases in Japan and to evaluate information on resources to support the daily and social life in the CPGs.

## Materials and Methods

### Setting in Japan

The Rare/Intractable Diseases Act leads to changes in the research framework ^(18)^. Consequently, close collaboration between the Ministry of Health, Labour and Welfare (MHLW) and the Japan Agency for Medical Research and Development (AMED) began to promote research on rare and intractable diseases in Japan ^(18)^. At present, AMED also promotes international cooperation to advance medical research ^(24)^ and leads core programs, such as the Initiative on Rare and Undiagnosed Diseases (IRUD) ^(25), (26)^. Moreover, Orphanet Japan, supported by AMED, cooperates with these programs and works toward improving the visibility of rare diseases ^(27)^. The MHLW develops and disseminates diagnostic criteria and evidence-based CPGs ^(18)^. Furthermore, the Medical Information Distribution Service (Minds), a guideline clearinghouse supported by the MHLW, is devoted to the preparation, evaluation, and dissemination of information ^(19)^.

### Definition of rare diseases

Rare and intractable diseases in Japan are characterized by the following: (1) unknown etiology, (2) lack of effective treatment, (3) low prevalence, and (4) long-term medical care needed ^(14), (18), (28)^. As for “designated rare/intractable diseases” eligible for the medical subsidy system, the additional requirements included (5) a disease prevalence of less than 0.1% in Japan and (6) establishment of indicators for diagnosis ^(14), (28), (29)^. We identified 327 out of the 331 designated rare/intractable diseases ^(30)^ in 2018 as “rare diseases,” of which the number of patients in Japan was less than 50,000. Thus, we excluded four designated rare/incurable diseases: ulcerative colitis, Parkinson’s disease, systemic lupus erythematosus, and Sjögren’s syndrome. The disease with less than 50,000 patients is eligible for orphan designation in pharmaceutical regulation ^(31), (32)^, translating to a rate of approximately 4 in 10,000 people ^(33)^. In the EU, any diseases affecting fewer than 5 in 10,000 people are considered rare ^(33)^. We compiled a list of disease names, including 327 diseases ^(30), (34)^, for this study.

### Eligibility criteria

The inclusion criteria were as follows: (1) the title or the table of contents of the document matching with our list of disease names, (2) published from January 2015 to August 2018, and (3) full text available in Japanese. The exclusion criteria were as follows: (1) focus on specific life events, (2) pertaining to only specific conditions such as impairment and complications, (3) translated version of foreign CPGs, (4) the only description of tests or diagnosis, and (5) no description of a term indicating literature search or certainty of evidence.

### Information sources and searches

Our study mainly targeted CPGs developed just after the enforcement of the Rare/Intractable Diseases Act in 2015. In general, government research groups in Japan generally take approximately 2 years to develop CPGs from launch to publication ^(35)^. Hence, we set our search period to August 2018. We searched PubMed and three Japanese databases, i.e., Toho University and Japan Medical Abstracts Society CPGs information database ^(36)^, the National Diet Library Online ^(37)^, and the Minds Guideline Library ^(38)^ (from January 2015 to August 2018). In addition, we manually searched the fiscal years 2014 to 2017 reports of the Policy Research Project for Rare/Intractable Diseases in the MHLW Grants System ^(39)^ (from April 2015 to August 2018) and the Japan Intractable Diseases Information Center ^(30)^ (from January 2015 to August 2018). The search was conducted from September 1 to September 24, 2018.

We searched the Japanese databases using the following terms: “Shinryo (clinical practice),” “Chiryou (treatment),” “Gaidorain (guideline),” “Shishin (guideline),” “Tebiki (guide, guidance),” “Manyuaru (guide, guidance),” “Shinryo-gaido (guide for clinical practice, guidance, consensus),” “Chiryou-gaido (guide for treatment),” and “Sansho-gaido (reference guide).” Two reviewers independently screened the documents based on the eligibility criteria. When the screening results of the two did not match, a consensus was reached through discussion.

### Assessment of methodological quality

The Appraisal of Guidelines for Research and Evaluation (AGREE) II instrument ^(40)^ was used to assess the eligible CPGs while referring to the AGREE Reporting Checklist ^(41)^. The AGREE II instrument allows us to comprehensively assess the transparency and methodological rigor of the CPG development process. AGREE II provides a standardized framework consisting of 23 items over 6 domains: *Scope and Purpose*, *Stakeholder Involvement*, *Rigor of Development*, *Clarity of Presentation*, *Applicability*, and *Editorial Independence*. Each item was rated on a 7-point scale (1, strongly disagree, to 7, strongly agree). If an item for a particular guideline was “not described,” the item was scored as 1. The appraisers rated the overall quality of each guideline (1-7) and whether they recommended its use. The domain score was calculated as follows: (obtained score − minimum possible score)/(maximum score − minimum possible score).

The eligible CPGs were assessed by a multidisciplinary team consisting of seven healthcare providers: a family physician, an occupational therapist, a mental health social worker, a midwife, an acupuncturist, a gastroenterological surgeon, and a registered nurse. The first appraiser was TU, and the second was one of the six selected randomly. All appraisers were trained using the AGREE online tool. To eliminate the concern about generous ratings because of the CPGs for rare diseases ^(42)^, the appraisers assessed and discussed the two non-covered CPGs in this study. Two appraisers first assessed each CPG independently, then discussed and exchanged opinions, and finally reassessed it independently. We determined the median, 25th percentile, and 75th percentile for the domain and item scores.

### Evaluation of information on non-medical resources in CPGs

Information on non-medical resources, which support daily and social life, in the CPGs was evaluated as follows: (1) An expert panel was organized. The panel consisted of a physician certified by the Japanese Society of Public Health, a designated physician for rare diseases, and a registered nurse with experience in caring for patients living with rare disease, and they consulted a medical social worker as appropriate. (2) The expert panel identified three documents for extracting the keywords: the Rare/Intractable Diseases Act ^(14)^ and two relevant government reports of the Act ^(15), (16)^. (3) Based on the three documents, the expert panel extracted and agreed on five keywords, including “Intractable Disease Consultation Support Center,” “Japan Intractable Disease Information Center,” “Patient Association,” “Medical Subsidy System,” and “Designated Rare/Intractable Diseases,” which indicate information on non-medical resources to support patients living with rare diseases in their community (Table 1). (4) Two appraisers examined the main text of each CPG, whether the keywords were described at the time of the AGREE II assessment. When the results of the two did not match, a consensus was reached through discussion. (5) We used content analysis ^(48), (49)^ to evaluate information on non-medical resources in the main text of CPGs. We counted the number of CPGs that included the predetermined keywords. The percentage was then calculated with the eligible CPGs for the AGREE II assessment as the denominator and the number of CPGs described keywords as the numerator.

**Table 1.** Five Keywords Indicating Information on Non-medical Resources.

Keywords	Explanations
Intractable Disease Consultation Support Center	The centers offering support to address psychological and social issues, which are in each prefecture to support their daily lives and to improve the quality of life for patients living with rare/intractable disease in the community ^(43)^. The centers offer services such as consultation and information provision on formal support programs available to patients living with rare/intractable disease, promoting social engagement, organizing employment support through counseling for adaptation to the workplace and arranging employment opportunities, coordinating of peer supports, and holding workshops for the patients and their family ^(43), (44)^.
Japan Intractable Diseases Information Center	Information support by a MHLW project, which is the reference source on rare/intractable diseases online. The website is designed to allow access equally to stakeholders on information about incurable diseases. The website contains information such as various Acts or Programs for rare/intractable diseases, designated rare/intractable disease projects, national research projects, clinical trials, centers of experts, patient associations, practice guidelines, and knowledge for the public ^(45)^.
Patient Association	Peer support. Informational, educational, psychological, and practical support based on extremely similar illness experiences ^(46)^.
Medical Subsidy System	Financial support as regulated by the Rare/Intractable Disease Act. Patients with rare/intractable disease are required to submit a clinical survey form, which is marked by a designated rare/intractable disease clinician, to the local healthcare center in each prefecture. When the criteria for financial support are met, the patient is eligible for the support ^(47)^.
Designated Rare/Intractable Diseases	A term indicates the diseases subjected to the medical subsidy system regulated by the Rare/Intractable Disease Act ^(47)^. This term can serve as a signpost to make healthcare providers aware of non-medical resources available to patients living with rare/intractable diseases. The term also indicates the organization of national specialized research groups for each disease to overcome it ^(28), (45), (47)^.

MHLW, Ministry of Health, Labour and Welfare; Rare/Intractable Disease Act, Act on Medical Care for Patients with Rare/Intractable Diseases (Act No.50 of 2014).

## Results

### Selection of CPGs

**Figure 1.**
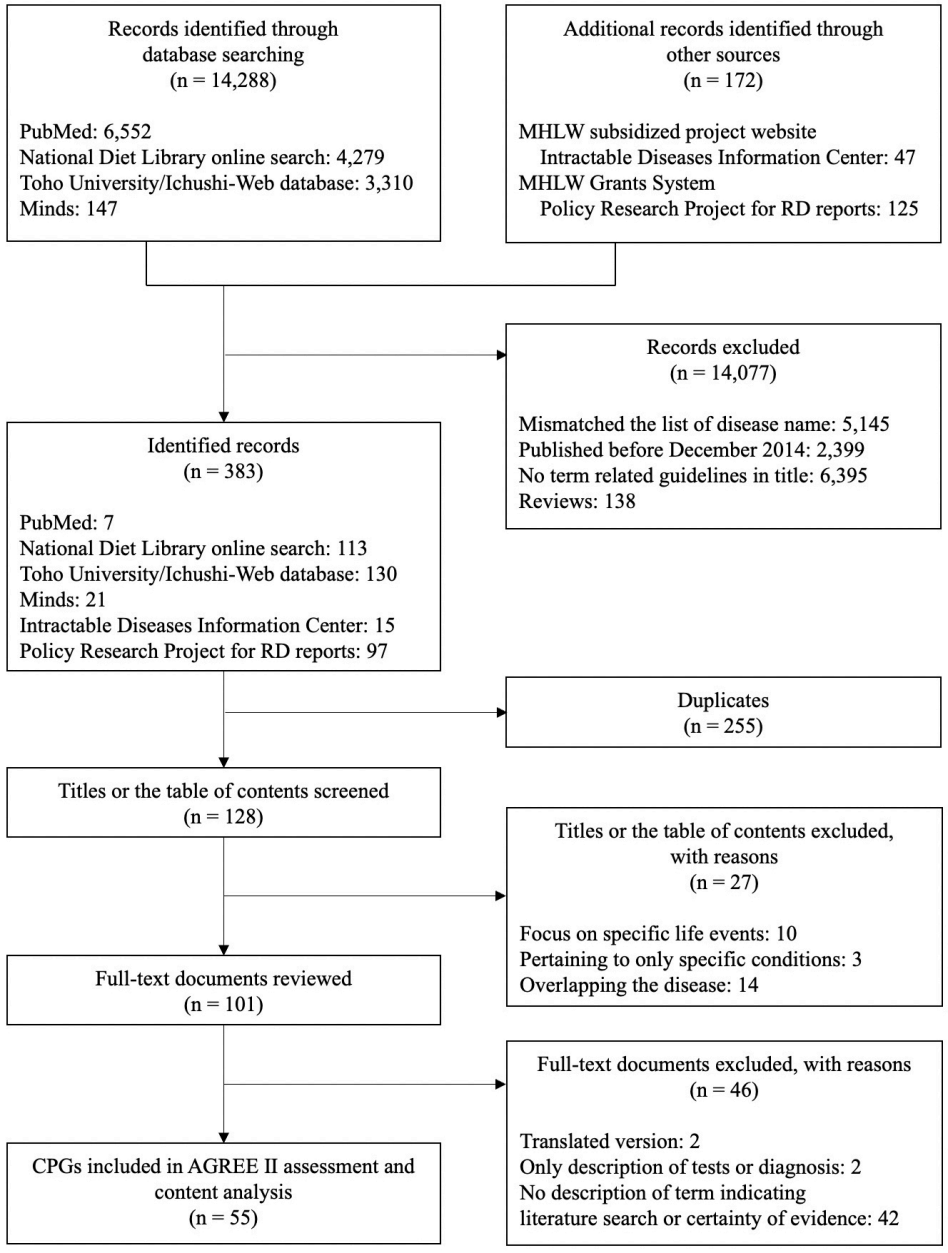
Flow diagram of CPG selection. CPG, clinical practice guideline; Toho University/Ichushi-Web database, the Toho University and Japan Medical Abstracts Society clinical practice guidelines database; Minds, the Minds Guideline Library; MHLW, Ministry of Health, Labour and Welfare; Policy Research Project for RD reports; the reports of the Policy Research Project for Rare/Intractable Diseases; AGREE, Appraisal of Guidelines for Research and Evaluation.

The search yielded 14,460 documents. After excluding documents with a mismatch between the disease names in the title or the table of contents and the list of disease names and duplicates, 128 documents remained. Finally, we selected 55 CPGs (including 90 diseases) that met the eligibility criteria (Figure 1). A total of 37 guidelines (including 37 diseases) were identified by matching titles to the list of disease names and 18 (including 53 diseases) by reconfirming the table of contents or headings. Table 2 shows that half of the selected CPGs were published in 2017. Except one CPG, the guideline development entity was jointly organized by the policy research project groups supported by the MHLW and related medical societies.

**Table 2.** Characteristics of the Included CPGs (*n* = 55).

Characteristics	CPGs included
Published year	
2015	5
2016	6
2017	32
2018 (until August)	12
Edition	
First edition	29
Revised edition	26

### Assessment using AGREE II instrument

Table 3 shows the median scores for the 6 AGREE II domains and 23 items of the 55 CPGs. The median scores for domain *Stakeholder Involvement*, *Rigor of Development*, *Applicability*, and *Editorial Independence* were lower than 50%. The lowest median score among the six domains was that for the* Rigor of Development* (28%). Item 7 in *Rigor of Development* had a median of 5 and a 25th percentile of 1. Regarding the reporting criteria ^(41)^ included in item 7, 21 CPGs described the full search strategy and 37 described the named electronic database or evidence source where the search was performed.

**Table 3.** Median, 25th, and 75th Percentile for 6 Domain Scores and 23 Item Scores of AGREE II Assessment in the Included CPG (*n* = 55).

Domains/items	Domain score^*,†^	Item score^‡,†^
Scope and Purpose	69% (47%, 78%)	
1. The overall objective(s) of the guideline is (are) specifically described.		5 (3, 6)
2. The health question(s) covered by the guideline is (are) specifically described.		5 (5, 6)
3. The population (patients, public, etc.) to whom the guideline is meant to apply is specifically described.		5 (4, 6)
Stakeholder Involvement	42% (25%, 61%)	
4. The guideline development group includes individuals from all relevant professional groups.		4 (3, 5)
5. The views and preferences of the target population (patients, public, etc.) have been sought.		1 (1, 3)
6. The target users of the guideline are clearly defined.		6 (3, 6)
Rigor of Development	28% (17%, 53%)	
7. Systematic methods were used to search for evidence.		5 (1, 6)
8. The criteria for selecting the evidence are clearly described.		3 (1, 5)
9. The strengths and limitations of the body of evidence are clearly described.		2 (2, 4)
10. The methods for formulating the recommendations are clearly described.		2 (1, 3)
11. The health benefits, side effects, and risks have been considered in formulating the recommendations.		3 (2, 5)
12. There is an explicit link between the recommendations and the supporting evidence.		4 (3, 6)
13. The guideline has been externally reviewed by experts prior to its publication.		2 (1, 5)
14. A procedure for updating the guideline is provided.		1 (1, 3)
Clarity of Presentation	69% (42%, 86%)	
15. The recommendations are specific and unambiguous.		5 (3, 6)
16. The different options for management of the condition or health issue are clearly presented.		5 (3, 6)
17. Key recommendations are easily identifiable.		5 (5, 7)
Applicability	31% (13%, 46%)	
18. The guideline describes facilitators and barriers to its application.		2 (2, 5)
19. The guideline provides advice and/or tools on how the recommendations can be put into practice.		3 (2, 5)
20. The potential resource implications of applying the recommendations have been considered.		3 (2, 5)
21. The guideline presents monitoring and/or auditing criteria.		2 (1, 3)
Editorial Independence	38% (17%, 50%)	
22. The views of the funding body have not influenced the content of the guideline.		3 (2, 5)
23. Competing interests of guideline development group members have been recorded and addressed.		3 (2, 4)
Overall Guideline Assessment		
1. Rate the overall quality of this guideline.		3 (2, 5)

AGREE, Appraisal of Guidelines for Research and Evaluation; CPG, clinical practice guideline * Each domain score is calculated between 0% and 100%. † Median (25th percentile, 75th percentile) ‡ Each item is rated on a 7-point scale (1, strongly disagree, to 7, strongly agree).

Eleven CPGs (20%) described how the views and preferences of the target population were considered, including nine with the participation of patient representatives. Among the 35 CPGs (64%) with a stated target user, 18 mentioned only medical doctors as audience members. The median score for item 9 was 2. Most of the CPGs only described the level of evidence and the grade of recommendation corresponding to the study design. The recommendations in 12 CPGs (22%) were expert consensus that considered the reviews of case reports and case series due to a lack of randomized controlled trials and high-quality observational studies for specific clinical questions. A total of 45 CPGs (82%) mentioned whether the treatment was covered by insurance.

### Evaluation of information on non-medical resources

Table 4 shows the 5 keywords in 55 CPGs, which indicate information on non-medical resources to support patients living with rare diseases in their community. A total of 41 CPGs (75%) included at least 1 of the 5 keywords, although 14 CPGs included none. The three CPGs (5%) included the* Intractable Disease Consultation Support Center* for multiple sclerosis, spinocerebellar degeneration, and systemic amyloidosis (Supplemental Material Table S1). The description of the* Japan Intractable Disease Information Center*, *Patient Association*, * Medical Subsidy System*, and *Designated Rare/Intractable Diseases* was 12 (22%), 17 (31%), 21 (38%), and 36 (65%), respectively.

**Table 4.** Five Keywords in 55 CPGs.

Keywords	*n* = 55	Percentage
Intractable Disease Consultation Support Center	3	5%
Japan Intractable Diseases Information Center	12	22%
Patient Association	17	31%
Medical Subsidy System	21	38%
Designated Rare/Intractable Diseases	36	65%

CPG, clinical practice guideline

## Discussion

We assessed the methodological quality of existing CPGs for rare diseases in Japan using the AGREE II instrument and found that the median of the four domain scores was below 50%. Of the six domains, *Rigor of Development* had the lowest median score. To explore clues for providing pragmatic guidance, we evaluated five keywords as information on non-medical resources. Three quarters of the CPGs included at least one of the five keywords, while one quarter of the CPGs included none. Three CPGs (5%) included the* Intractable Disease Consultation Support Center*.

With the methodological quality assessment using the AGREE II instrument of the CPGs, Cassis et al. ^(50)^, who evaluated guidelines for inherited neurometabolic disorders, reported that the mean score of *Stakeholder Involvement*, *Rigor of Development*, *Applicability*, and *Editorial Independence* was lower than 50%. Our results showed a similar trend with the results of the previous study. However, Sasaki et al. ^(51)^, who evaluated CPGs for priority common diseases in Japan, reported that the tendency for the median score of the four domains was likely to be low. Guideline development groups may have a common challenge to be trustworthy CPGs, regardless of the quantity or quality of clinical epidemiological evidence available. Existing methodological approaches have evolved, aiming to develop scientifically valid and trustworthy CPGs and to reduce various biases that could arise from the development process of CPGs ^(52)^. In any development process of CPGs, it is desirable to utilize the applicable parts of existing methodological approaches for the CPGs to be trustworthy for users.

Among all six domains in AGREE II, the median for *Rigor of Development* (composed of items 7-14) was lower than that previously reported ^(50)^ (Supplemental Material Table S2). Among the eight items in this domain, the scores of items 11 and 12, the consideration of benefits and harms and the link between recommendations and evidence, were lower in this study than in the previous one ^(50)^ (Supplemental Material Table S3). The previous study was limited to English documents ^(50)^ and included the Scottish Intercollegiate Guideline Network (SIGN) ^(53)^ and the National Institute for Health and Care Excellence (NICE) ^(54)^ in electronic databases in their search strategy. The SIGN methodology ^(53)^ and NICE methodology ^(54)^ use a consensus-based approach, among others, to formulate recommendations when little or no evidence exists. They also revealed that the scores on items 7-10 widely varied across documents. This observation is consistent with our results. Items 7 and 8 were related to the search and selection of the study. Even if no eligible clinical epidemiological research is identified, systematic reviews are an essential process to be a trustworthy CPG ^(52)^. The CPG was required to explicitly present specific clinical questions that could not be addressed with current evidence ^(55)^. We expect to describe the search strategies and study selection processes to provide reproducible systematic reviews. On the other hand, items 9 and 10 are related to translation evidence to recommendations ^(3), (56)^. The low scores on the items may closely reflect the methodological challenges. The RARE-Bestpractices Working Group indicated that case series and case reports would be informative ^(56)^. In addition, the group proposed and evaluated the framework in the development of CPGs for rare diseases such as registry analysis and clinical experience-based opinions ^(6)^. Moreover, several groups are actively exploring approaches to extracting and synthesizing evidence or knowledge from non-epidemiological research. A working group examined the confidence in findings from systematic reviews of qualitative research ^(57)^, and another investigated the methods to integrate different types of knowledge from various sources such as etiology and the context of care ^(58)^. These approaches may contribute to the advancement of frameworks for the development of CPGs for rare diseases.

Each keyword as information on non-medical resources was insufficiently described across the CPGs (Table 4). Patients living with rare diseases have suffered from conflicting information provided by healthcare practitioners unfamiliar with their disease conditions ^(59)^. One of the most delicate aspects facing the patients and healthcare practitioners is patient acceptance of the diagnosis, progression, and illness ^(60)^. Healthcare practitioners should play an important role in recognizing the illnesses faced by individual patients in the context of their lives and in providing social support ^(61), (62)^. Most of the patients and their families, to live in the community, have had to bear the considerable burdens in identifying their barriers and coordinating the care and support on their own ^(22)^. Therefore, providing information on non-medical resources to support the patients may lead to advocating for psychosocial care in the usual clinical practice and could be the clues to provide pragmatic guidance for users.

Among the five keywords representing information on non-medical resources, the three CPGs, including *Intractable Disease Consultation Support Center*, were for multiple sclerosis, spinocerebellar degeneration, and systemic amyloidosis. These diseases were targeted at the nationwide epidemiological survey for intractable diseases supported by the Ministry of Health and Welfare in the 1970s to accumulate knowledge on addressing the psychological burden, social issues, and medical disease management ^(28), (63)^. A distinctive role of the intractable disease consultation support centers is to deliver practical work-life support through career guidance and coordination (Table 1). Cobb and Hamera illustrated that the negative effects of the loss of work, position, and role in society that accompanied the progression of the disease through multiple timely interviews ^(64)^. Ponzio et al. mentioned that the current workers with multiple sclerosis had higher social care needs for workplace adaptation through a questionnaire survey related to unmet care needs ^(60)^. The decline in physical function not only affects employment, finances, and social engagements ^(22)^ but also deepens the sense of loss of independence and autonomy in their daily life ^(21)^. The psychosocial challenges faced by people living with a rare disease are numerous and seriously affect their self-esteem and autonomy ^(22)^. Enhancing patient outcomes demands providing the best medical care and addressing psychosocial challenges tailored to the complex context individually ^(61)^. Healthcare practitioners could benefit the patients living with rare diseases by using their expertise to provide information on appropriate support considering patient values and circumstances.

The approach using non-medical resources in clinical practice is closely in line with the social prescribing ^(65), (66), (67), (68)^, which is developed for the General Practitioner system within the United Kingdom. The social prescribing approach has important implications for this study. One of the key elements of the approach is that healthcare practitioners connect the patients who need support with non-medical resources to the link workers ^(69), (70)^. The approach is strategically designed into the healthcare system as part of usual clinical practice ^(71), (72)^. Although appropriately connecting with community health care is a desperate issue for most patients with rare diseases and their families in complex circumstances, they face difficulties in accessing professionals to coordinate care and support ^(22)^. The approach can contribute to the formation of care and support pathways between clinical and community settings ^(73)^. CPGs for rare diseases, including information on non-medical resources, may act as a bridge across the fragmented pathways between health and social care ^(16), (22), (23), (74)^. By embedding the formation of the pathways in clinical practice, we can find a possible solution for the implementation of a comprehensive support system to avoid the neglect of patients living with rare diseases from social security schemes.

This study has some limitations. First, we did not organize an additional research plan for the assessment using the Appraisal of Guidelines Research and Evaluation-Recommendation Excellence (AGREE-REX) ^(75)^, which emphasized the clinical applicability, values and preferences, and implementability, after it was released. We used the AGREE II instrument and the AGREE Reporting Checklist as assessment tools with careful consideration to standardize the assessment procedure. The AGREE II instrument focuses on development processes and forms, particularly on structured and rigorous methodologies. Furthermore, the present findings were based only on the literature that we accessed. It would be preferable to contact the guideline development group to clarify the actual process and their views.

### Conclusions

We assessed the methodological quality of the existing CPGs for rare diseases in Japan using the AGREE II instrument and found that the domain score of *Stakeholder Involvement*, *Rigor of Development*, *Applicability*, and *Editorial Independence* was low. The methodological challenges may have resulted in the insufficient description of items regarding the translation evidence to recommendations in the *Rigor of Development*, which requires the approaches to apply the CPG development for rare diseases. Moreover, the keywords determined by the expert panel as information on non-medical resources were insufficiently described across the CPGs. This indicates that the information is advocative as clues to provide pragmatic guidance, particularly for rare diseases with limited medical evidence.

## Article Information

### Conflicts of Interest

Dr. Nakayama reports personal fees from Pfizer Japan Inc., MSD K.K., Otsuka Pharmaceutical Co., Chugai Pharmaceutical Co., Dentsu Co., Takeda Pharmaceutical Co., Janssen Pharmaceutical K.K., Boehringer Ingelheim International GmbH, Eli Lilly Japan K.K., Baxter, Alexion, Mitsubishi Tanabe Pharma Corporation, Novartis Pharma K.K., Allergan Japan K.K., Maruho Co., Ltd., research grants from HANSHIN Dispensing Holding Co., Ltd., Nakagawa Pharmacy Co., Ltd., and Konica Minolta, Inc. All of them were outside the submitted work.

### Sources of Funding

This work was supported by the Health and Labour Sciences Research Grants from the Ministry of Health, Labour and Welfare, Japan [H30-Iryo-Shitei-023]. The funding sources had no role in the collection, analysis, or interpretation of the data or in the decision to submit the article for publication.

### Acknowledgement

The authors would like to express their appreciation to Ayako Kumamura, a medical social worker at Kyoto University Hospital, for her professional and practical advice. We would also like to thank Linda Cassis and Elisenda Cortès-Saladelafont for providing information that was not included in their articles.

### Author Contributions

TU: conceptualization, data curation, formal analysis, investigation, methodology, project administration, resources, validation, visualization, writing - original draft, and writing - review and editing. YT: conceptualization, formal analysis, methodology, project administration, validation, visualization, writing - original draft, and writing - review and editing. HY: investigation, methodology, validation, and visualization. YN: investigation, methodology, and validation. T Ohura, T Okura, and YM: investigation, methodology, and validation. MH and HK: investigation and validation. TS: validation and writing - original draft. TN: conceptualization, funding acquisition, methodology, project administration, supervision, validation, visualization, writing - original draft, and writing - review and editing.

### Approval by Institutional Review Board (IRB)

Not applicable.

## Supplement

Supplementary File
