# Supplementary material for: Evaluation of Clinical Practice Guidelines for Rare Diseases in Japan
Source: JMA J. 2022 Sep 30;5(4):460–70. doi: 10.31662/jmaj.2022-0094 (PMC9646292; doi:10.31662/jmaj.2022-0094)
Supplement: Supplementary file 1 — Supplementary File [file 2433-3298-5-4-0460-s001.pdf]

Table S1. Five keywords in each CPG (n = 55)

	Intractable Disease Consultation Support Center	Japan Intractable Diseases Information Center	Patient Association	Medical Subsidy System	Designated Rare/Intractable Diseases
CPGs (n)	3	12	17	21	36
1	X	X	X	X	X
2	X	X			
3	X		X	X	X
4-7		X	X	X	X
8		X	X		X
9		X	X		
10		X		X	X
11-12		X			X
13		X			
14-19			X	X	X
20			X		X
21-22			X		
23-30				X	X
31-41					X
42-55					

(1) CPG1: CPG for Multiple sclerosis/neuromyelitis optica; (2) Amyloidosis; (3) Spinocerebellar degeneration; (4) Vascular anomalies; (5) Autoinflammatory disease; (6) Pulmonary hypertension; (7) Prion disease; (8) Adrenoleukodystrophy; (9) Subacute sclerosing panencephalitis; (10) Progressive multifocal leukoencephalopathy; (11) Familial hypercholesterolemia; (12) Sarcoidosis; (13) Stevens-Johnson syndrome/toxic epidermal necrolysis; (14) ANCA-associated vasculitis, (15) Cystinosis; (16) Acute encephalopathy in children; (17) Dementia; (18) Pulmonary veno-occlusive disease/pulmonary capillary hemangiomatosis; (19) Chronic thromboembolic pulmonary hypertension; (20) Dystonia; (21) Wilson's disease; (22) Biliary atresia; (23) Vasculitis syndrome; (24) Thrombotic thrombocytopenic purpura; (25) Autoimmune hemorrhaphilia XIII; (26) Feurofibromatosis 1; (27) Diseases targeted for newborn screening; (28) Congenital heart disease in adults; (29) Acquired idiopathic generalized anhidrosis; (30) Idiopathic pulmonary fibrosis, (31) Alport’s syndrome; (32) Rapidly progressive glomerulonephritis; (33) Autoimmune hepatitis; (34) Non-IgE-mediated gastrointestinal food allergy; (35) Adult-onset Still's disease; (36) Dermatomyositis/polymyositis; (37) Pseudoxanthoma elasticum; (38) Allied Hirschsprung’s disease; (39) Pompe disease; (40) Mucopolysaccharidosis type 2; (41) Pemphigoid; (42) Werner's syndrome; (43) Nephrotic syndrome; (44) Polycystic kidney disease; (45) IgA nephropathy; (46) Inflammatory bowel disease; (47) Vasculitis / vasculopathy; (48) Primary biliary cholangitis; (49) Acquired hemophilia A; (50) Congenital diaphragmatic hernia; (51) Generalized scleroderma; (52) Epilepsy; (53) Giant infantile hepatic hemangioma; (54) Generalized pustular psoriasis; (55) Portal hypertension.

Table S2. Summary of the six domain scores of AGREE II assessment

	Cassis et al.* 2000 to 2015 (*n* = 55)		The present study 2015 to August 2018 (*n* = 55)	
Domains	mean ± SD	median	mean ± SD	median
1. Scope and Purpose	78% ± 12%	81%	64% ± 23%	69%
2. Stakeholder Involvement	48% ± 22%	44%	45% ± 24%	42%
3. Rigor of Development	48% ± 20%	46%	38% ± 27%	28%
4. Clarity of Presentation	74% ± 16%	78%	64% ± 24%	69%
5. Applicability	39% ± 15%	38%	31% ± 22%	31%
6. Editorial Independence	41% ± 35%	46%	38% ± 24%	38%

Each domain score was calculated between 0% and 100%.

* Data were derived from Cassis et al. report [Cassis L, Cortes-Saladelafont E, Molero-Luis M, et al. Review and evaluation of the methodological quality of the existing guidelines and recommendations for inherited neurometabolic disorders. Orphanet J Rare Dis. 2015;10:164[(50)]].

Table S3. Summary of the eight item scores in domain 3 (Rigor of Development) of AGREE II assessment

Domain 3: Rigor of Development	Cassis et al.* 2000 to 2015 (n = 55)		The present study 2015 to August 2018 (n = 55)	
Items	mean ± SD	median	mean ± SD	median
7. Systematic methods were used to search for evidence.	4.4 ± 2.0	4.5	3.9 ± 2.4	5
8. The criteria for selecting the evidence are clearly described.	3.3 ± 2.0	3	3.4 ± 2.2	3
9. The strengths and limitations of the body of evidence are clearly described.	3.7 ± 1.8	3.5	3.0 ± 1.9	2
10. The methods for formulating the recommendations are clearly described.	3.7 ± 1.9	3.5	2.7 ± 1.8	2
11. The health benefits, side effects, and risks have been considered in formulating the recommendations.	5.4 ± 1.2	6	3.6 ± 1.8	3
12. There is an explicit link between the recommendations and the supporting evidence.	5.6 ± 1.2	6	4.2 ± 1.7	4
13. The guideline has been externally reviewed by experts prior to its publication.	2.6 ± 2.1	1.5	2.9 ± 1.9	2
14. A procedure for updating the guideline is provided.	2.3 ± 1.7	1.5	2.4 ± 2.1	1

Each item was rated on a 7-point scale (1: strongly disagree to 7: strongly agree).

* As the data were not included by Cassis et al. in their report [Cassis L, Cortes-Saladelafont E, Molero-Luis M, et al. Review and evaluation of the methodological quality of the existing guidelines and recommendations for inherited neurometabolic disorders. Orphanet J Rare Dis. 2015;10:164[(50)]], the authors provided the data on demand from us. We thank the authors, who provided us with this information.
